# Solubility of Ca_5_(PO_4_)_3_OH in the System Ca(OH)_2_-H_3_PO_4_-H_2_O at 5, 15, 25, and 37 °C*

**DOI:** 10.6028/jres.081A.017

**Published:** 1977-04-01

**Authors:** H. McDowell, T. M. Gregory, W. E. Brown

**Affiliations:** Institute for Materials Research, National Bureau of Standards, Washington, D.C. 20234

**Keywords:** Dissolution, hydroxyapatite, ion pairs, solubility, solubility isotherms, solubility product, thermal coefficient of solubility, thermodynamics, tooth mineral

## Abstract

Solubility isotherms of hydroxyapatite, Ca_5_(PO_4_)_3_OH (OHAp), prepared by titrating a boiling aqueous suspension of Ca(OH)_2_ with 0.5 *M* H_3_PO_4_, were determined in the ternary system Ca(OH)_2_–H_3_PO_4_–H_2_O at 5, 15, 25, and 37 °C in the pH range 3.7–6.7 by equilibration with dilute H_3_PO_4_ solutions. The solubility product *K_s_*, determined as a function of temperature by a generalized least-squares procedure from 41 experimental points, is given by the equation

$$\log\;K_{s}=-8219.41/T-1.6657-0.098215T.$$

The values of *K_s_* and its dispersion at 25 and 37 °C are 3.04 (0.25) and 2.35 (0.27) × 10^−59^. There is a maximum in *K_s_* near 16 °C, which may be due to the form of temperature dependence found earlier for the stability constants of the ion pairs ${\text{CaH}}_{2}{\text{PO}}_{4}^{+}$ and ${\text{CaHPO}}_{4}^{0}$.

The relative positions of the isotherms show that OHAp has a negative thermal coefficient of solubility. Thermodynamic functions for the dissolution of the salt are reported.

The solubility data previously reported by others for OHAp at 25 °C were reviewed. The solubility products obtained by three of these investigators were comparable with our value of 3.0 × 10^−59^; their data were reevaluated by the method described here. We conclude that the best value for the solubility product at 25 °C is 4.7 (2.0) × 10^−59^.

## 1. Introduction

Hydroxyapatite, Ca_5_(PO_4_)_3_OH, is an important component of bones and teeth and of many industrial products. Despite the profusion of papers on the chemistry of hydroxyapatite [1–5]^1^ the exact solubility of this salt is still questioned. Its existence as a compound with a fixed crystalline form having a well-defined solubility product has been disputed [2, 4]. This is due, in part, to the fact that its preparations vary in composition. Recently it has been found that this salt exists in two modifications, one monoclinic, the other (statistically) hexagonal [6]. Some of the difficulties encountered in solubility measurements on hydroxyapatite and the experimental principles that can be used to overcome them are described in greater detail elsewhere [7]. Much of the uncertainty regarding the solubility of the salt also stems from a combination of factors related to the difficulty of preparing pure samples and to the fact that it usually precipitates in a very finely divided state so that contamination by adsorption and occlusion of impurities readily occurs. Solubility measurements on this salt are difficult because its relatively low solubility and its slow rate of equilibration from supersaturation make the establishment of equilibrium uncertain. The solubility data previously reported on this salt have all been for 25 °C.

In making solubility measurements on hydroxyapatite workers have used two approaches. On the one hand, precipitates formed from aqueous solutions at low temperatures have been separated and redispersed directly in an equilibrating medium [1]. On the other, the solubility measurements have been made following treatment of the precipitate by exposure to elevated temperatures [5]. Both procedures have yielded apatites with a constant solubility product over a wide range of aqueous compositions, although the two types of treatments give materials with somewhat different values for the solubility product. Here we report the results of solubility measurements at 5, 15, 25, 37 °C on a precipitate that was not heat treated (except for drying at 110 °C). A precipitated product was used because it should be more representative of the apatites found in biological and other natural systems. The data were used to calculate solubility products and thermodynamic functions for this salt taking into account ion pair formation. In addition to the work reported here, we have re-examined solubility data obtained at 25 °C in other studies [8–10] of hydroxyapatites prepared in a manner similar to the one used here. The adjustment procedure [5, 11–15] used in treating the data is unusually rigorous and provides a superior assessment of the reliability of the derived constants.

## 2. Experimental Methods and Materials

### 2.1 Preparation of Materials

Hydroxyapatite was prepared by titrating a boiling aqueous suspension of Ca(OH)_2_ with 0.5 *M* H_3_PO_4_. The Ca(OH)_2_ suspension was prepared by adding 194 g of CaO, made by calcination of CaCO_3_ for 24 hours at 1000 °C, to seven liters of freshly boiled distilled water. The titration was carried out in a 15 liter, Teflon-coated,^2^ stainless steel pot equipped with a gold plated stirrer, a reflux condenser protected from the atmosphere by a CO_2_-absorbing trap, and ports for introducing N_2_ and the titrant. The H_3_PO_4_ solution was added to the stirred suspension at a rate of 1 mL/min until a Ca/P ratio of 1.70 was reached. After the titration was complete, boiling and stirring were continued for two days. The solid was then allowed to settle and the supernatant liquid was removed by siphoning. Five liters of freshly boiled distilled water were then added to the reaction vessel and the resulting suspension was boiled and stirred for two days to remove the Ca(OH)_2_. The supernatant was then siphoned off and the washing process was repeated three times with distilled water and finally with 0.001 *M* H_3_PO_4_. The slurry was then transferred to a three liter Erlenmeyer flask and dried in an oven at 110 °C under a stream of dry N_2_.

Doubly crystallized phosphoric acid hemihydrate, 2H_3_PO_4_·H_2_O, and freshly boiled distilled water were used to prepare a 1 *M* stock solution. The H_3_PO_4_ solutions used for the titration and equilibrations were prepared by diluting the stock solution with freshly boiled distilled water.

Examination of the solid with a petrographic microscope gave a mean refractive index of 1.636; this agrees with the values usually found for precipitated Ca_5_(PO_4_)_3_OH [5]. The material was free of extraneous phases but the crystallites were too small to be resolved. The infrared spectrum of the material indicated that it was crystalline and essentially free of carbonate. The BET surface area^3^ of the solid was 16.7 m^2^/g. Chemical analysis yielded: Ca, 39.25 percent and P, 18.13 percent. (Theoretical: Ca, 39.89 percent and P, 18.50 percent.) From the analytical results the molar Ca/P ratio of the solid was 1.67_3_ with a standard error of 0.042.

### 2.2. Equilibration Experiments

To each of a series of glass-stoppered bottles, 4 g of hydroxyapatite were added and washed several times with small volumes of the H_3_PO_4_ solution with which the salt was to be equilibrated. About 125 mL of this acid were then added and the bottle was sealed with paraffin wax and rotated end-over-end at 7 rpm in a thermostatted water bath held to within 0.1 °C of the selected temperature. In preliminary experiments lasting up to 21 days, it was shown that equilibrium was attained within 7 days. Thereafter, solutions equilibrated for at least 7 days were filtered, using a syringe equipped with a millipore filter, and the pH values and calcium and phosphorus concentrations of the filtrates were determined.

### 2.3. Analyses

Calcium, phosphorus, and pH were determined using the methods and instruments described in earlier publications [11, 15]. The estimated errors for Ca (1.5%), P (1.0%), and pH (0.015) guided the assignment of weights (sec. 5).

## 3. Calculations

Details of the method used for the calculation of solubility products and ion pair association constants by generalized least squares have been reported previously [11]. The specific equations and conditions needed to describe equilibrium systems containing solid Ca_5_(PO_4_)_3_OH are given here. Curly brackets will be used to denote equation, section and table numbers used in Reference [11]. The purpose of these calculations was to obtain the solubility product, *K_s_*, of Ca_5_(PO_4_)_3_OH as a function of temperature, allowing for the presence of ion pairs, and to compute the adjusted values of the observations. The values used for the association constants of the ion pairs ${\text{CaHPO}}_{4}^{0}$ and ${\text{CaH}}_{2}{\text{PO}}_{4}^{+}$ are those given in table {12}^4^. In 30 systems (out of 41 reported here) the weighted sum of squares of residuals of four observations per system (concentration of total calcium, Ca; and total phosphorus, P; initial phosphoric acid concentration, P_0_; and pH) was minimized subject to three condition functions: saturation with respect to Ca_5_(PO_4_)_3_OH, electroneutrality, and congruent dissolution (sec. 5.2) of the salt. In the remaining 11 systems (3 at 5 °C, 5 at 15 °C, and 3 at 37 °C) the condition of congruent dissolution was omitted and the variable P_0_ was accordingly not adjusted for these systems (see discussion). The solubility product *K_s_* was computed by the least squares estimation of the coefficients *A_j_* in the expression ln *K_s_* = *A*_1_/*T* + *A*_2_ + *A*_3_*T*, where *T* is the temperature in Kelvins (eq {16}).

### 3.1. Balance Equations, Solubility Product, and Activity Coefficients

The equations of mass balance are given by eqs {1} to {4}. Activity coefficients (Debye-Hückel) and ionic strength are defined in eqs {10} and {11a}. (All quantities were calculated on the molar basis.) The ionic activity product is defined for all Ca_5_(PO_4_)_3_OH as

$$K_{\text{HA}}={\left({\text{Ca}}^{2+}\right)}^{5}{\left({\text{PO}}_{4}^{3-}\right)}^{3}({\text{OH}}^{-})$$ (1)

where the first factor (activity of ionic calcium) and the second (activity of orthophosphate ion) are defined as follows:

$$({\text{Ca}}^{2+})=(\text{Ca}-\tau )/g_{\text{Ca}}$$ (2a)

$$({\text{PO}}_{4}^{3-})=(P-\tau )k_{3}/(H^{+})N.$$ (2b)

The quantities *g*_Ca_, *k*_3_, and *N* are defined in the equations of [11] already referred to; (H^+^) = 10^−pH^, and *τ* is the sum of ion pair concentrations, eq {8}. The apparent solubility product *K*′_HA_ was calculated taking *τ* to be zero.

### 3.2. Condition Functions, Parameters, and Standard Deviation

The condition functions imposed on the systems reported here have been enumerated above. The electroneutrality condition is given by eq {14}. The other two conditions are defined here as:

1. Saturation with respect to Ca_5_(PO_4_)_3_OH
  $${\left({\text{Ca}}^{2+}\right)}^{5}{\left({\text{PO}}_{4}^{3-}\right)}^{3}({\text{OH}}^{-})-K_{s}=O,$$ (3)
  where the activities are given by eqs (2a) and (2b), and by (OH^−^) = ***k****_w_*/(H^+^), where ***k****_w_* is the dissociation constant of water;
2. Congruent dissolution of Ca_5_(PO_4_)_3_OH
  $$\text{Ca}/(P-P_{0})-R=0,$$ (4)
  where *R* is the expected value of the ratio, 5/3.

Since the ion pair association constants were not subject to adjustment in these calculations, the number of constants is just one (*K_s_*); the number of adjustable parameters (*p*) is one or three corresponding to separate (*l*n *K_s_ = A*_2_) or combined (*l*n *K_s_ = A*_1_/*T* + *A*_2_ + *A*_3_*T*) data adjustments, respectively (see sec. {3.5b}). The standard deviation is given by eq {22} with the denominator (degrees of freedom) in that equation replaced by Σ*_t_*(2*N_t_* + 3*M_t_*) − *p*. Here *N_t_* and *M_t_* denote the number of systems at temperature *t* constrained by 2 and 3 conditions, respectively.

## 4. Results

### 4.1. Solubility and Solubility Product

The compositions of saturated Ca_5_(PO_4_)_3_OH solutions at 5, 15, 25, and 37 °C are presented in tables 1 through 4. At all temperatures except 25 °C there were one or more systems whose dissolution ratios (col. 5) were inconsistent with congruent dissolution (expected value = 5/3). These data, however, yielded values (eq (1)) of the apparent solubility product, *K*′_HA_ (col. 6) which clearly belonged to the population being sampled. These points were therefore included in the analysis (see sec. 5.4). The values of *K*_HA_ computed with the data shown in these tables and with the assumption of ion pairs are shown in the last column of the tables as $K_{\text{HA}}^{0}$. It is noteworthy that in the more acid solutions, values of *K*′_HA_ that might be suspected to represent outliers on the high side are better behaved when ion pairs are taken into account. [Table t2-jresv81an2-3p273_a1b] [Table t3-jresv81an2-3p273_a1b]

The data adjustments as found for the combined data are summarized in tables 5 through 8. Values of the ionic strength, *μ*, shown in column 5, indicate the broad range of solution compositions included in the investigation. The data segregated in the lower portion of the tables were adjusted without the congruent dissolution condition (eq (4)) and hence the initial acid concentration, P_0_, of these systems was ignored in the adjustment. The ratios Ca/(P − P_0_) for the other systems computed with the untruncated adjusted data and subsequently rounded to 6 digits are uniformly equal to 1.66667. Similarly, the adjusted values of *K*_HA_ at temperature *T* in the last columns are identical (to five digits or better) to the value of the least-squares constant *K*_s_, which is given to sufficient accuracy by eq (5):

$$\log\;K_{s}=-8219.41/T-1.6657-0.098215\;T.$$ (5)

[Table t6-jresv81an2-3p273_a1b] [Table t7-jresv81an2-3p273_a1b]

Values of the solubility product, its logarithm, and the associated standard errors (eq {25}) at the four temperatures are listed in table 9. (The standard error of the adjustment, *s*, is discussed in sec. 5.1.)

**Figure 1:**
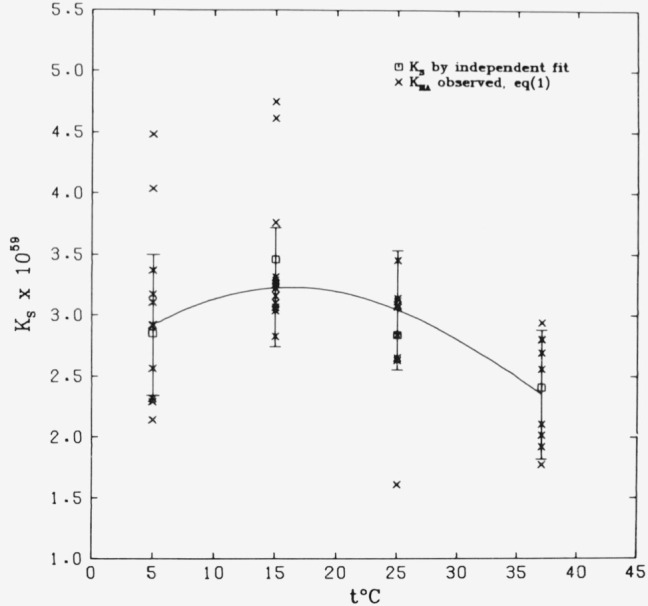
Variation in the solubility product of hydroxyapatite as a function of temperature. The curve represents eq (5); the 95 percent confidence intervals come from table 9.

Figure 1 illustrates the temperature dependence of the solubility product. The smooth curve represents eq (5) (95% confidence intervals come from table 9). The preliminary values of *K_s_* from separate adjustments at the four temperatures (table 12) are shown for comparison. The values plotted in the figure as experimental points are those listed in column 7 of tables 1 through 4.

**Figure 2:**
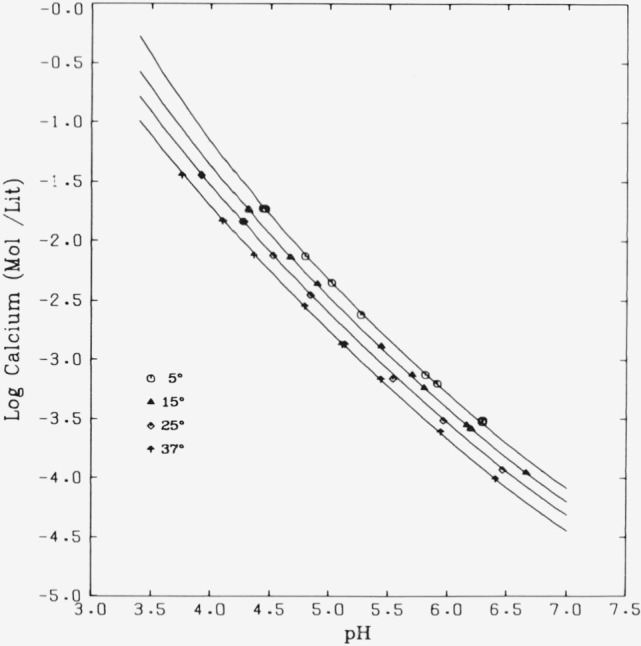
Solubility isotherms (log calcium concentration versus pH) for hydroxyapatite in the system *Ca*(*OH*)_2_–*H*3*PO*_4_–*H*_2_*O* at 5, 15, 25, and 37 °C.

The solubility isotherms for Ca_5_(PO_4_)_3_OH are plotted in figure 2 in terms of log (total Ca concentration) against pH. The smooth curves were generated as described in reference [21]. The experimental points are from tables 1 through 4. The adjusted values of the observations (not indicated in the figure) lie on the curves. The relative positions of the curves and corresponding data sets indicate that the solubility of hydroxyapatite decreases with increasing temperature even though it can be seen from figure 1 that the solubility product does not decrease monotonically with temperature. This type of behavior has been previously established for CaHPO_4_·2H_2_O [11], CaHPO_4_ [15], and *β*-Ca_3_(PO_4_)_2_ [13].

### 4.2. Ion Pairs

The concentrations of the ion pairs ${\text{CaHPO}}_{4}^{0}$ and ${\text{CaH}}_{2}{\text{PO}}_{4}^{+}$ in saturated solutions of Ca_5_(PO_4_)_3_OH at 25 °C are given in table 10. These values^5^ were computed in the adjustment procedure, making use of the known values [11] of the corresponding stability constants. The percentage of bound calcium (sum of ion pair conc. × 100/total Ca conc.) shown in column 4 is seen to increase markedly in the more acid solutions due to the rapid increase in the concentration of the ion ${\text{CaH}}_{2}{\text{PO}}_{4}^{+}$ (col. 3) with decreasing pH. The corresponding effect on the values of the “observed” solubility product, columns 6 and 7 of tables 1 through 4, is quite evident. (Values of the ion pair concentrations at the other temperatures are similar to those shown in table 10.)

### 4.3. Thermodynamic Quantities

The dissolution of Ca_5_(PO_4_)_3_OH may be described by the reaction

$${\text{Ca}}_{5}{\left({\text{PO}}_{4}\right)}_{3}\text{OH}=5{\text{Ca}}^{2+}+3{\text{PO}}_{4}^{3-}+{\text{OH}}^{-}.$$ (6)

The quantities Δ*G*°, Δ*H*°, and Δ*S*° associated with the dissolution reaction were calculated from eq (5) for log *K_s_* following the procedure of eqs {27–29}. The results, along with their standard errors, are set out in table 11.

## 5. Discussion

### 5.1. Standard Deviation, Weights and Goodness of Fit

Table 12 summarizes the results of preliminary adjustments that were carried out to examine the temperature dependence of *K*_HA_ and to test for uniformity of variance (*s*^2^, eq {22}) among the four temperature sets. The variances listed there were homogeneous by Bartlett’s test [16]. These results in table 12 were obtained with the following assignment of errors: Ca, 2.0 percent; P, 1.5 percent; P_0_, 1.0 percent; pH, 0.020 (absolute). The corresponding weights (eq {12}, with *s*_0_ = 1 × 10^−4^) were then utilized in the final adjustment of the combined data. The overall variance was found to be *s*^2^ = 1.13 × 10^−8^, based on 109 degrees of freedom, *f*(sec. 3.2). The critical values of $\chi_{109}^{2}$ (95%), 82.0 and 139.8, nicely bracket the value of *f*, thus confirming the adequacy of the weights used and establishing the goodness of fit of the adjustment. The standard deviation is then 1.06 × 10^−4^, and the 95 percent confidence interval for *s* is (0.94 < *s* < 1.22 × 10^−4^).

### 5.2. Solubility Isotherms

The data reported in tables 1 through 4 were obtained from phosphoric acid solutions of widely varying initial concentration (less than 1 to over 50 mM) equilibrated with pure solid Ca_5_(PO_4_)_3_OH. It is evident from the plots in figure 2 that the experimental points at each temperature are well represented by the corresponding calculated curves. The latter resulted from the adjustment procedure which for all points assumes (i) equilibrium (saturation) and (ii) electroneutrality (i.e., all species known and accounted for, including ion pairs). In addition a third requirement, that of congruent dissolution (no extraneous solid phases are formed), was imposed on 30 out of the total of 41 systems. Since the remaining 11 systems, adjusted with only conditions (i) and (ii), are not distinguishable in figure 2 from those subjected to all three conditions, it appears that the smaller set of systems was indeed saturated with respect to Ca_5_(PO_4_)_3_OH. The question of stoichiometry will be dealt with in more detail below (sec. 5.4). It suffices to emphasize here that failure to exhibit congruent dissolution is not in itself a sufficient reason to doubt the attainment of the equilibrium state.

The success of the adjustment procedure applied to these data is further illustrated by reference to tables 5 through 8. The standard errors (in parentheses) are generally within 1 percent of the adjusted observations. The individually computed solubility products, *K*_HA_ (eq (1)), shown in column 7, using the adjusted observations, are identical to the corresponding least-squares value of *K_s_* (eq (5); table 9). Both the dissolution ratio, column 6 (where applicable) and the electroneutrality condition (not shown) were satisfied to better than 1 ppm. Additional insight as to the validity of the adjustment under the given conditions may be gained from the following results (not reported). The magnitudes of the largest relative adjustments (residual/observed) were: Ca, 0.046 at 37 °C; P, 0.025, and P_0_, 0.023 at 15 °C. The size of the maximum residual in pH was 0.036 at 15 °C. The sizes of the corresponding standardized residuals (residual/S.E. of residual) were 2.4, 1.8, 3.3, and 1.8.

### Effect of Temperature on the Solubility of Ca_5_(PO_4_)_3_OH

The relative positions of the solubility isotherms depicted in figure 2 indicate, as mentioned in section 4, that the solubility of this preparation of hydroxyapatite has a negative temperature coefficient. It is of interest to examine this behavior under the condition of uniform initial acid concentration. Since at each of the four temperatures there were values of the adjusted initial acid (P_0_, tables 5 through 8) near 1 mM and 10 mM, it was feasible to compute the conditional values [17] of Ca, P, and pH, and the corresponding concentrations of the ion pairs, at these two rounded values of P_0_. The results are given in the following table:

**Table:** 

°C	P_0_ = 1 mM			P_0_ = 10 mM		

	Ca	P	pH	Ca	P	pH
5	0.761	1.457	5.82	7.22	14.33	4.82
15	.750	1.450	5.68	7.17	14.30	4.68
25	.742	1.445	5.55	7.14	14.28	4.55
37	.734	1.440	5.40	7.10	14.26	4.40

Although the concentration differences between temperatures are small, on the order of the standard errors, the trend is consistently toward lower concentration with increasing temperature. The percentage of bound calcium, 100*τ*/Ca (not listed), shows a marked reduction of over 50 percent between 5 and 37 °C; fom 2.7 to 1.2 (P_0_ = 1 mM), and from 8.6 to 3.9 (P_0_ = 10 mM).

### 5.3. Solubility Product Temperature Dependence and Models

Equation (5) for log *K_s_* derived by the least squares adjustment possesses a maximum near 16 °C, as seen in figure 1. Maxima have also been shown to occur in the vicinity of 20 °C for the solubility products of CaHPO_4_·2H_2_O [11] and Ca_3_(PO_4_)_2_ [13]. In the case of CaHPO_4_·2H_2_O it was argued that the maximum is a possible consequence of the thermodynamic properties of the solid. In the present work the influence of the ion pair assumption on the nature of the temperature dependence of the solubility product of Ca_5_(PO_4_)_3_OH was investigated. A least squares adjustment was accordingly carried out without the assumption of the presence of ion pairs. The equation corresponding to eq (5) gave the following values of *K_s_* at the four experimental temperatures: 4.2, 4.0, 3.6, and 2.9 × 10^−59^. The standard deviation of the adjustment was 1.08 × 10^−4^, with a slightly wider confidence interval than was reported in section 5.1 for the model with ion pairs included. It is evident that the maximum in *K_s_* for Ca_5_(PO_4_)_3_OH can be ascribed, at least in part, to the nature of the temperature dependence of the ion pair stability constants as reported in [11].

### 5.4. Stoichiometry. Potential Diagrams

**Figure 3:**
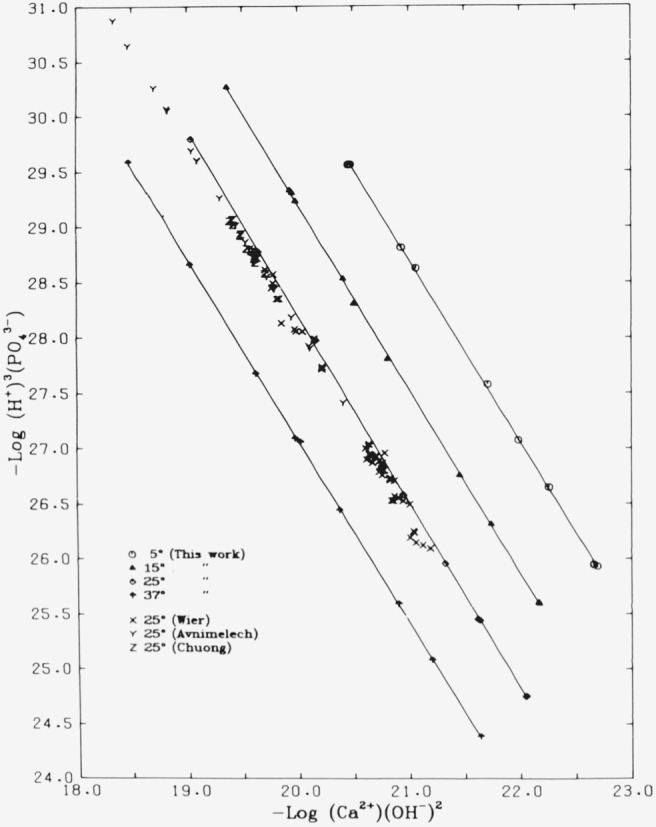
“Potential plot” (−log *H*_3_*PO*_4_ activity versus −log *Ca*(*OH*)_2_ activity) for hydroxyapatite at 5, 15, 25, and 37 °C. Solid lines are least-square fits of data in tables 1–4.

**Figure 4:**
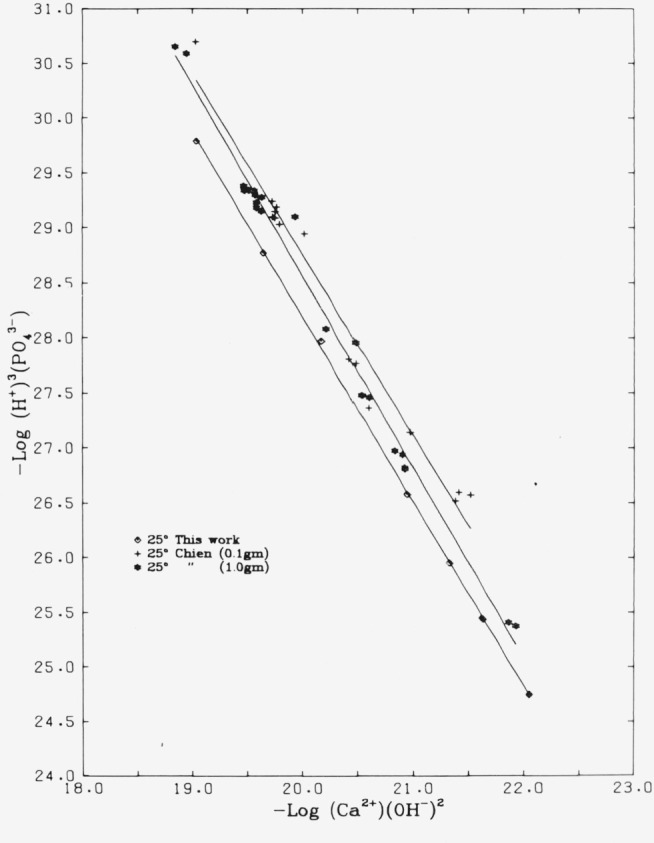
“Potential plot” for hydroxyapatite at 25 °C comparing our data with those of Chien et al. [8] for the “autoclaved” sample.

Equilibrium between a solid calcium phosphate and an aqueous solution, and the stoichiometry of the saturating solid, may be demonstrated by a “potential diagram” [18], in which the coordinates are defined as − log[(Ca^2+^)(OH^−^)^2^] and $-\log[{\left(H^{+}\right)}^{3}\left({\text{PO}}_{4}^{3-}\right)]$. These are linearly related to the chemical potentials of the solution components Ca(OH)_2_ and H_3_PO_4_. The saturation condition in such a diagram is defined by a straight line, whose slope is the negative of the Ca/P ratio in the solid. Figures 3 and 4 show the data of tables 1 through 4, as well as results at 25 °C reported by some other workers, plotted in this manner. The lines in figure 3 were fitted by least squares to the data of tables 1 through 4 only. The slopes at 5, 15, 25, and 37 °C are −1.643, −1.666, −1.682, and −1.645, respectively. When the fitting was done with a forced common slope, the value obtained was −1.660. (The standard error of the slope in all cases is less than 0.01). These values compare favorably with the values for the Ca/P ratio, 1.67_3_, found by analysis of the solid (sec. 2.1) and the theoretical value for Ca_5_(PO_4_)_3_OH, 1.667 (5/3). It is evident that the subset of 11 systems that failed to show congruent dissolution (sec. 5.2) cannot be distinguished in these plots from those that did satisfy that condition; thus all of the data represent solutions saturated with respect to a solid of very nearly the theoretical stoichiometry of 5/3. The results of the other workers shown in the figures are discussed in the following section.

### 5.5. Comparison With Reported Data

Avnimelech, Moreno, and Brown [9] reported solubility measurements at 25 °C on a sample of hydroxyapatite prepared by a method very similar to the one described in this paper. Their data (20 points), included in figure 3, very closely parallel our results at this temperature except that stoichiometric dissolution (i.e., a Ca/P ratio of 5/3) was the exception. This is not surprising because the objective of their research was to show that the surface Ca and P contribute significantly to the apparent stoichiometry of the overall dissolution reaction even though the stoichiometry of the final equilibrium reaction was shown to have the stoichiometry 5/3 (see their fig. 3). They reported a *K*_HA_ of 6.32 (2.1) × 10^−59^ (arithmetic mean) and a value of −1.66 (.02) for the slope of the line (not shown in our fig. 3); the Ca/P ratio of their solid was 1.69. We have recalculated the solubility product using the adjustment procedure described in this report; the resulting value of *K_s_* is 5.23 (0.41) × 10^−59^.

Chuong [10] reported a series of 18 measurements on the four component system Ca(OH)_2_ – H_3_PO_4_ – H_2_O – HCl at 25 °C to ascertain the effect of varying the solid-to-solution ratio; his solid was a portion of the same preparation that was used in the present study. His results, shown in figure 3, cover only a narrow range of the potential diagram, but are seen to be consistent with the data of Avnimelech et al. There was a slight trend in *K*_HA_ with increasing HCl concentration (mean values were 6.2, 5.4 and 5.0 × 10^−59^ at HCl concentrations of 0.70, 1.0 and 10.0 mmol/L, respectively), but no trend was observed at fixed acid concentration with solid-to-solution ratios varying from 0.2 to 2.0 g/100 mL. An overall estimate of the solubility product was not reported; we have computed a least-squares value of 5.36 (0.19) × 10^−59^ from his data.

Wier, Chien, and Black [8] have reported an extensive series of hydroxyapatite solubility measurements (112 equilibrations at 25 °C) in which the ratio of solid-to-solution varied from 0.1 to 10.0 g/100 mL. They reported their solubility constants as *pK*_HA_ based on the formula Ca_10_(PO_4_)_6_(OH)_2_ which we have converted into *K*_HA_ values based on the formula Ca_5_(PO_4_)_3_OH. The hydroxyapatite used in their equilibrations was a commercial product. In five of their six series of equilibrations, this hydroxyapatite had been boiled in water for 24 hours; in the sixth series, the hydroxyapatite had been treated with 1 *N* NH_4_Cl solution at 120 °C in an autoclave for 50 days, replacing the NH_4_Cl solution every 4 to 6 days. The purpose of the latter treatment was to further stabilize the hydroxyapatite and thereby to demonstrate the existence of a form of hydroxyapatite that is less soluble than the material used in their first five series of experiments. The first five series included 44 equilibrations with solid-to-solution ratios between 0.5 and 10.0. This set of points is included in figure 3 (Wier). Although there is considerable scatter, these points are comparable with the others at 25 °C in the figure. Wier et al. reported a solubility product equal to 5.0 × 10^−59^ for 32 systems of this set (solid-to-solution ratio of 1.0 g/100 mL). Making use of their measurements on all 44 systems, we derive a value of *K_s_* equal to 6.18 (0.70) × 10^−59^.

In addition to the above values, Moreno et al. [5] reported two values for *K_s_* at 25 °C, one for a preparation heated in steam at 1000 °C, 3.7 (0.5) × 10^−58^, and one heated in air at the same temperature, 2.5 (0.4) × 10^−55^. The latter is four orders of magnitude larger than the value reported here and reflects changes in the structure induced by heating in air at 1000 °C. Hydroxyapatite is unstable when heated in air at 1000 °C and tends to disproportionate into Ca_3_(PO_4_)_2_ and Ca_4_(PO_4_)_2_O. Moreno et al. reported evidence for the presence of *β*-Ca_3_(PO_4_)_2_ in the air-heated sample. For this reason, very little weight should be given to the *K_s_* of the air-heated sample.

Hydroxyapatite is believed to be the stable phase when heated at 1000 °C in a steam atmosphere. As noted below, the *K_s_* for the steam-heated sample is significantly higher than the value reported here; thus, the steam-heat treatment may have altered the structure of the hydroxyapatite in some way.

Clark [1] has reported a value of similar magnitude, 20 × 10^−59^, for a sample of hydroxyapatite prepared by precipitation at 90 °C.

The values of *K_s_* that we derived from the data of the above mentioned investigators are summarized in table 13 along with the value obtained from our data. Clearly the values of Clark and of Moreno et al. are substantially different from the other four values. When the average of the latter four values is taken with weights inversely proportional to the squares of the given standard errors, we derive a value of 4.7 (0.1) × 10^−59^ as the most probable value for the solubility product constant at 25 °C of hydroxyapatite that has not received thermal treatment. In view of the spread of these four values, an estimate of 2.0 × 10^−59^ is a reasonable value of the uncertainty to be assigned to the derived *K_s_*.

Wier et al. gave two values for the *K*_HA_ of the autoclaved hydroxyapatite, one obtained with 0.1 g of solid per 100 mL of solution, 5.6 × 10^−61^ (15 points) and the other with 1.0 g of solid per 100 mL, 2.2 × 10^−60^ (22 points). These data are displayed in the potential plot of figure 4; our results at 25 °C are again shown for comparison. Because there appear to be substantial differences among the three sets, we have reevaluated the data of Wier et al. for the sixth series with the procedures and constants used by us so as to put all the data in figure 4 on a comparable basis. (Some uncertainty was encountered in estimating the ionic strength; unspecified amounts of HCl had been added to the equilibrating solutions. We used the apparent electroneutrality imbalance, always positive in these solutions, as a measure of chloride concentration.) We obtained *K_s_* values of 0.46 (0.14) and 1.95 (0.48) × 10^−60^, respectively, for the autoclaved samples. Ninety-five percent confidence intervals for the solubility products of the 0.1 and 1.0 gm/100 mL sets are, respectively, 0.17 < *K_s_* < 0.75 × 10^−60^, and 0.97 *< K_S_* < 2.93 × 10^−60^. It may be remarked that a value of 1 × 10^−60^ corresponds to a substantially lower Gibbs energy, about 2 kcal per mol, in the autoclaved sample as compared to the sample used by us and the one used by them in their first five series of equilibrations.

We concur with the principal conclusion of Wier et al., that hydroxyapatite may have a variable solubility product. Variability in the solubility of hydroxyapatite is indicated by the study of Moreno et al. [5], on synthetic hydroxyapatite, the studies of Patel et al. [19], on enamel, and that of Mattamal and Brown [20], on bone. Furthermore, it is known that hydroxyapatites have variable indices of refraction, unitcell dimensions, impurity contents, degrees of disorder in the OH positions, and may possibly vary in their Ca and OH contents. Thus, there is ample basis for ascribing variable solubility to hydroxyapatites. The question then arises as to whether the low values reported by Wier et al. for the autoclaved sample represent solubility products for more completely annealed and stabilized hydroxyapatite or are a consequence of experimental errors. This warrants examination of the experimental technique which produced these results.

Wier et al. [8] used an equilibration apparatus consisting of two chambers separated by a cellulose acetate dialysis membrane. The same initial equilibration solution was present in both chambers, but the solid was present in only one and the solution for analysis was taken from the other. Thus, for the solution in the sample chamber to be in equilibrium with the solid, it is necessary for the solution in the first chamber to be saturated with respect to the solid, and for the two solutions to be in equilibrium across the membrane. Equilibrium across the membrane requires that the chemical potentials (or activities) of both Ca(OH)_2_ and H_3_PO_4_ must be the same in the two solutions. The use of a membrane undoubtedly slows the approach to equilibrium, particularly if the membrane possesses some permselectivity. In the present case one might anticipate that the membrane would be somewhat permselective to cations in accord with the usual character of cellulose acetate membranes; the data for the autoclaved sample do provide an indication of this. The ΔCa/ΔP ratios in many of the equilibrations fall in a range much higher than the theoretical value, 1.67, thus indicating that the phosphate ions passed through the membrane less readily than the calcium ions and that trans-membrane equilibrium with respect to the component H_3_PO_4_ may not have been attained. If so, this may account for the relatively large variation in their solubility products. We believe this is the main reason for the difference in the values 0.046 (0.014) and 0.195 (0.048) × 10^−59^ for *K_s_* obtained with the two solid-to-solution ratios, 0.1 and 1.0, respectively. No such variation with solid-to-solution ratio was observed by Chuong. Furthermore, the lower value for *K*_HA_ obtained by them with the smaller solid-to-solution ratio would be in accord with the view that these equilibrations had not reached saturation.

The true minimum value for the solubility product of hydroxyapatite continues to be of considerable interest because it would represent the most appropriate standard state for this solid, and it would be the structural condition toward which biological and other hydroxyapatites would tend to convert. It remains to be seen if a hydroxyapatite with *K*_HA_ significantly lower than the mean value reported here, 4.7 × 10^−59^, can exist.

## Figures and Tables

**Table 1:** Solubility of hydroxyapatite at 5 °C Unadjusted quantities

Initial Acid P_0_ *M* × 10^3^	Composition of equil. soins			$\frac{\text{Ca}}{P-P_{0}}$	*K*′_HA_ × 10^59^	*K°*_HA_ × 10^59^
	pH	Ca *M* × 10^3^	P *M* × 10^3^			

0.846	5.91_2_	0.630	1.19_5_	1.81	3.73	3.17
1.04_4_	5.81_3_	.748	1.45_5_	1.82	3.03	2.56
3.22	5.26_9_	2.40	4.75	1.57	2.80	2.14
6.22	5.02_2_	4.46	8.88	1.68	4.28	2.92
10.2_4_	4.79_8_	7.45	14.6_6_	1.69	3.87	2.32
26.0	4.45_9_	18.6	37.3	1.65	7.64	3.10
26.0	4.45_6_	18.7	36.7	1.75	7.11	2.92
26.0	4.44_0_	18.7	36.9	1.72	5.58	2.29
0.410	6.29_5_	0.307	0.544	*a*(2.29)	5.18	4.48
.410	6.29_4_	.302	.540	(2.32)	4.66	4.04
.410	6.29_0_	.297	.532	(2.44)	3.88	3.37

a Value inconsistent with congruent dissolution.

**Table 2 t2-jresv81an2-3p273_a1b:** Solubility of hydroxyapatite at 15 °C Unadjusted quantities

Initial Acid P_0_ *M* × 10^3^	Composition of equil.solns.			$\frac{\text{Ca}}{P-P_{0}}$	*K*′_HA_ × 10^59^	*K°*_HA_ ×10^59^
	pH	Ca *M* × 10^3^	P *M* × 10^3^			

1.04_4_	5.70_2_	0.751	1.47_7_	1.73	5.25	4.75
1.89	5.44_2_	1.31	2.59	1.87	5.22	4.61
6.25	4.90_0_	4.36	8.62	1.84	4.78	3.76
10.4_0_	4.67_1_	7.32	14.6_1_	1.74	4.33	3.10
25.9	4.31_4_	18.0	36.1	1.76	5.53	3.07
25.9	4.31_4_	18.3	36.3	1.76	5.94	3.29
25.9	4.30_7_	18.6	36.7	1.72	5.84	3.22
0.132	6.66_3_	0.111	0.177	(2.47)	3.47	3.25
.433	6.20_3_	.259	.460	(9.59)	3.27	3.04
.433	6.19_1_	.262	.472	(6.72)	3.04	2.83
.433	6.16_1_	.281	.521	(3.19)	3.57	3.31
.857	5.80_2_	.583	1.10_4_	(2.36)	3.45	3.16

**Table 3 t3-jresv81an2-3p273_a1b:** Solubility of hydroxyapatite at 25 °C Unadjusted quantities

Initial Acid P_0_ *M* × 10^3^	Composition of equil.solns.			$\frac{\text{Ca}}{P-P_{0}}$	*K*′_HA_ × 10^59^	*K°*_HA_× × 10^59^
	pH	Ca *M* × 10^3^	P *M* × 10^3^			

0.130	6.46_6_	0.117	0.203	1.60	2.77	2.63
.414	5.96_2_	.306	.599	1.65	2.81	2.65
.994	5.53_6_	.695	1.39_1_	1.75	1.72	1.61
5.11	4.84_2_	3.52	7.30	1.61	3.68	3.11
10.8_9_	4.52_3_	7.53	15.5_2_	1.63	4.01	3.07
20.9	4.27_1_	14.5	29.3	1.73	5.15	3.45
20.9	4.26_3_	14.5	29.5	1.69	4.61	3.08
53.0	3.92_2_	36.0	73.6	1.75	6.35	3.14
53.0	3.92_1_	35.5	72.4	1.83	5.70	2.84

**Table 4:** Solubility of hydroxyapatite at 37 °C Unadjusted quantities

Initial Acid P_0_ *M ×* 10^3^	Composition of equil.solns.			$\frac{\text{Ca}}{P-P_{0}}$	*K*′_HA_ × 10^59^	*K°*_HA_ × 10^59^
	pH	Ca *M* × 10^3^	P *M* × 10^3^			

0.994	5.43_6_	0.686	1.40_3_	1.68	3.02	2.81
1.92	5.12_9_	1.35	2.69	1.75	3.07	2.80
1.92	5.10_7_	1.34	2.75	1.61	2.21	2.02
10.8_9_	4.35_7_	7.56	15.6_3_	1.59	2.44	1.92
20.9	4.10_1_	14.7	29.8	1.65	3.01	2.11
52.9	3.75_4_	35.6	73.9	1.70	3.30	1.78
0.140	6.40_4_	0.0980	0.178	(1.32)	2.86	2.69
.433	5.94_0_	.245	.471	(6.45)	3.13	2.94
4.21	4.79_4_	2.83	5.66	(1.95)	2.92	2.56

**Table 5:** Solubility of hydroxyapatite at 5 °C Adjusted quantities

Initial Acid P_0_ *M* × 10^3^	Composition of equil.solns.			*μ* × 10^3^	$\frac{\text{Ca}}{P-P_{0}}$	*K*_HA_ × 10^59^
	pH	Ca *M* × 10^3^	P *M* × 10^3^			

0.834 (7)*b*	5.898 (7)	0.641(5)	1.219(10)	1.94	*a*1.67	2.92
1.023 (8)	5.806 (7)	.777(6)	1.489(12)	2.33	1.67	2.92
3.255 (26)	5.293 (7)	2.385(19)	4.686(38)	6.98	1.67	2.92
6.201 (50)	5.019 (7)	4.499(36)	8.900(72)	12.94	1.67	2.92
10.26 (8)	4.814 (7)	7.404(59)	14.70(12)	20.94	1.67	2.92
26.02 (21)	4.455 (7)	18.64(15)	37.20(30)	50.26	1.67	2.92
25.93 (21)	4.457 (7)	18.58(15)	37.07(30)	50.10	1.67	2.92
25.99 (21)	4.456 (7)	18.62(15)	37.16(30)	50.21	1.67	2.92
c*	6.272 (8)	0.301(4)	0.546(7)	0.939	*	2.92
*	6.276 (8)	.299(4)	0.541(7)	.932	*	2.92
*	6.283 (8)	.295(3)	0.533(7)	.920	*	2.92

a Machine value = 5/3 to seven significant figures.

b Standard error in the last digit(s) calculated by eq {26}.

c P_0_ not adjusted.

**Table 6 t6-jresv81an2-3p273_a1b:** Solubility of hydroxyapatite at 15 °C Adjusted quantities

Initial Acid P_0_ *M* × 10^3^	Composition of equil.solns.			*μ* × 10^3^	$\frac{\text{Ca}}{P-P_{0}}$	*K*_HA_ × 10^59^
	pH	Ca *M* × 10^3^	P *M* × 10^3^			

1.029 (8)	5.668 (6)	0.770(6)	1.491(12)	2.33	1.67	3.23
1.843 (15)	5.406 (6)	1.354(11)	2.656(21)	4.05	1.67	3.23
6.141 (50)	4.883 (6)	4.431(35)	8.810(71)	12.97	1.67	3.23
10.32 (8)	4.669 (6)	7.400(59)	14.76(12)	21.39	1.67	3.23
25.68 (21)	4.312 (6)	18.28(15)	36.64(30)	51.13	1.67	3.23
25.77 (21)	4.311 (6)	18.35(15)	36.78(30)	51.30	1.67	3.23
25.91 (21)	4.309 (6)	18.44(15)	36.97(30)	51.56	1.67	3.23
*	6.665 (8)	0.110(1)	0.178(2)	0.366	*	3.23
*	6.210 (7)	.255(3)	.465(6)	.802	*	3.23
*	6.201 (7)	.260(3)	.475(6)	.816	*	3.23
*	6.158 (7)	.283(3)	.519(7)	.883	*	3.23
*	5.805 (7)	.579(7)	1.109(14)	1.76	*	3.23

**Table 7 t7-jresv81an2-3p273_a1b:** Solubility of hydroxyapatite at 25 °C Adjusted quantities

Initial Acid P_0_ *M ×* 10^3^	Composition of equil. solns.			*μ* × 10^3^	$\frac{\text{Ca}}{P-P_{0}}$	*K*_HA_ × 10^59^
	pH	Ca *M* × 10^3^	P *M* × 10^3^			

0.1307(11)	6.475(6)	0.1179(8)	0.2014(16)	0.385	1.67	3.04
.4094(34)	5.958(6)	.3189(24)	0.6007(48)	.986	1.67	3.04
.978(8)	5.561(6)	.726(6)	1.414(11)	2.20	1.67	3.04
5.074(41)	4.832(6)	3.645(29)	7.261(58)	10.77	1.67	3.04
10.83(9)	4.516(6)	7.726(6)	15.46(12)	22.52	1.67	3.04
20.75(17)	4.259(6)	14.72(12)	29.58(24)	42.16	1.67	3.04
20.79(17)	4.258(6)	14.75(12)	29.65(24)	42.25	1.67	3.04
52.40(42)	3.913(6)	36.80(29)	74.48(60)	101.3	1.67	3.04
52.07(42)	3.916(6)	36.57(29)	74.02(60)	100.7	1.67	3.04

**Table 8:** Solubility of hydroxyapatite at 37 °C Adjusted quantities

Initial Acid P_0_ *M* × 10^3^	Composition of equil. solns.			*μ ×* 10^3^	$\frac{\text{Ca}}{P-P_{0}}$	*K*_HA_ × 10^59^
	pH	Ca *M* × 10^3^	P *M* × 10^3^			

0.977(8)	5.411(8)	0.718(6)	1.408(11)	2.16	1.67	2.35
1.900(15)	5.111(8)	1.375(11)	2.725(22)	4.11	1.67	2.35
1.911(15)	5.109(8)	1.383(11)	2.741(22)	4.13	1.67	2.35
10.88(9)	4.365(8)	7.719(62)	15.51(13)	22.57	1.67	2.35
20.91(17)	4.107(7)	14.73(12)	29.75(24)	42.41	1.67	2.35
52.49(43)	3.765(7)	36.42(29)	74.34(60)	101.4	1.67	2.35
*	6.390(9)	0.101(1)	0.175(2)	0.325	*	2.35
*	5.925(9)	.247(3)	.468(6)	0.764	*	2.35
*	4.789(8)	2.835(35)	5.655(70)	8.41	*	2.35

**Table 9:** Temperature dependence of the solubility product

log K*_s_* = −8219.41/*T*− 1.6657 −0.098215 *T* [eq (5)]		

°C	log K*_s_* + 59	K*_s_* × 10^59^

5	0.4656 (0.044)*a*	2.92 (0.30)
15	0.5090 (0.033)	3.23 (0.25)
25	0.4835 (0.036)	3.04 (0.25)
37	0.3716 (0.049)	2.35 (0.27)

a Standard error.

**Table 10:** Concentration of ion pairs and bound calcium at 25 °C

pH	[CaHPO_4_^0^] *M* × 10^3^	[CaH_2_PO_4_^+^] *M* × 10^3^	100 *τ*^a^/Ca

6.475	0.0008(<1)*b*	0.0001(<1)	0.79(1)
5.958	.0022(<1)	.0008(<1)	.93(1)
5.561	.0045(1)	.0039(1)	1.18(1)
4.832	.0166(2)	.077(1)	2.80(2)
4.516	.0294(4)	.282(5)	4.61(3)
4.259	.0473(6)	.820(13)	7.06(4)
4.258	.0473(6)	.823(14)	7.07(4)
3.913	.0900(12)	3.46(6)	12.5(1)
3.916	.0896(12)	3.43(6)	12.4(1)

a *τ* is sum of ion pair concentrations.

b Standard error in the last digit(s). See sec. 4.2.

**Table 11:** Thermodynamic quantities

°c	Δ*G*° kcal/mol	Δ*H*° kcal/mol	Δ*S°* cal*a*/mol·K

5	74.5 *b* (0.06)	2.8 (2.5)	−258 (10)
15	77.1 (0.04)	0.3 (1.2)	−267 (4)
25	79.8 (0.05)	−2.3 (1.1)	−276 (4)
37	83.2 (0.07)	−5.6 (2.9)	−286 (9)

a 1 cal = 4.1840 J.

b Standard error.

**Table 12:** Results of preliminary calculations

°C	Number of functions (*r*)	Number of points (*N*)	*f* c	Variance *s*^2^ × 10^8^	*K_s_* × 10^59^

5	3^a^ 2^b^	8 3	29	0.89	2.85
15	3 2	7 5	30	1.13	3.46
25	3	9	26	1.43	2.84
37	3 2	6 3	23	1.10	2.41

a Saturation, electroneutrality, and congruent dissolution.

b Saturation and electroneutrality.

c Degrees of freedom = Σ*Nr* − 1.

**Table 13:** Summary of adjusted values of the solubility product of Ca_5_(PO_4_)_3_OH at 25 °C.

Investigator	Ref.	*K_s_* × 10^59^	Comments
Moreno et al.	[5]	37.(5.)^a^	Heated in steam at 1000 °C.
Clark	[1]	20.^b^(3.)	Precipitated at 90 °C.
Wier et al.	[8]	6.18(0.70)	0.5 to 10.0 gm/100 mL.
Chuong	[10]	5.36(0.19)	0.2 to 2.0 gm/100 mL.
Avnimelech et al.	[9]	5.23(0.41)	
This work	–	3.04(0.25)	

a Estimated standard error of *K_s_*.

b Antilog of ½ times the mean of 27 values in table I of ref. [1]. (No allowance made for ion pairs).

## Footnotes

1 Figures in brackets indicate the literature references at the end of this paper.

2 In order to adequately describe materials and experimental procedures, it was occasionally necessary to identify commercial products by manufacturer’s name or label. In no instances does such identification imply endorsement by the National Bureau of Standards, nor does it imply that the particular product or equipment is necessarily the best available for the purpose.

3 The surface area measurement was made by Dr. W. V. Loebenstein, Dental & Medical Materials Section, NBS.

4 The ion pair ${\text{CaPO}}_{4}^{-}$ need not be considered for pH < 8.

5 The standard errors listed in the table were computed for each system with the aid of the covariance matrix (eq {A-10}) of the adjusted observations and the appropriate derivatives of equations {8–9} which define *τ* and the ion pair concentrations.
